# Unconventional Cadherin Localization in Honey Bee Gonads Revealed Through Domain-Specific *Apis mellifera* E- and N-Cadherin Antibodies Indicates Alternative Functions

**DOI:** 10.3390/insects3041200

**Published:** 2012-11-22

**Authors:** Mônica M. Florecki, Klaus Hartfelder

**Affiliations:** Departamento de Biologia Celular e Molecular e Bioagentes Patogênicos, Faculdade de Medicina de Ribeirão Preto, Universidade de São Paulo, Avenida Bandeirantes 3900, Ribeirão Preto 14049-900, SP, Brazil; E-Mail: mmflorec@gmail.com

**Keywords:** *Apis mellifera*, ovary, testis, cadherin, honeybee

## Abstract

As key factors in intercellular adhesion processes, cadherins play important roles in a plethora of developmental processes, including gametogenesis. In a previous study on cadherin localization in the gonads of honey bees, performed with heterologous pan-cadherin antibodies, we detected these proteins as (i) associated with cell membranes, (ii) as homogeneously distributed throughout the cytoplasm, and (iii) as nuclear foci in both somatic and germline cells, raising the possibility of alternative functions. To further investigate such unusual intracellular cadherin localization we produced specific antibodies against the N- and C-terminal domains of honey bee N- and E-cadherin. A 160 kDa protein was recognized by the E-cadherin antibodies as well as one of approximately 300 kDa from those raised against N-cadherin. In gonad preparations, both proteins were detected as dispersed throughout the cytoplasm and as nuclear foci in both germline and somatic cells of queen and worker ovarioles, as well as in the testioles of drones. This leads us to infer that cadherins may indeed be involved in certain signaling pathways and/or transcriptional regulation during gametogenesis. In late oogenesis stages, immunolabeling for both proteins was observed at the cell cortex, in conformity with a role in cell adhesion. In testioles, E-cadherin was seen in co-localization with fusomes, indicating a possible role in cyst organization. Taken together, the distribution of N- and E-cadherins in honey bee gonads is suggestive of alternative roles for cadherins in gametogenesis of both sexes.

## 1. Introduction

Honey bee queens are capable of laying 2,000 eggs and more per day. This is made possible through massive “parallel processing” in the nearly 200 ovarioles composing each ovary, whereby a constant flux of follicles is generated in the germarium. When reaching the vitellogenic growth phase, these then sequester the egg yolk precursor protein vitellogenin, produced in exceptionally high rates in the queen’s fat body and secreted into the hemolymph [1,2]. 

When viewed from the perspective of the *Drosophila* model on oogenesis in a polytrophic meroistic insect ovary, this would mean that in the honey bee ovary each germline stem cell would have to undergo at least five asymmetric divisions, thus generating five cystoblasts per day, and this continuously over months. Genuine germline stem cells have, however, not been unambiguously identified in honey bee ovarioles, even though histological preparations [3,4], actin and tubulin cytoskeleton analysis, and *vasa* mRNA localization [5,6,7] indicated that such cells may be housed as clusters within the extremely elongated terminal filaments of the ovarioles. This and other differences in comparison to the *Drosophila* model of oogenesis, as for instance the presence of actin in polyfusomes [6,8], raise the question as to how such functional differences may be built from a general *Bauplan* of a polytrophic meroistic ovary [9].

In the present study we focused on the expression and localization of cadherins, these being crucial molecules mediating cell-cell interactions between somatic and germline cells in insect and mammalian gonads of both sexes [10,11,12,13]. In *Drosophila*, germline stem cells are anchored through adherens and gap junctions to somatic cells (cap or hub cells) to their niches at the tip of ovarioles and testioles [14,15,16,17]. The importance of this niche architecture was denoted in expression studies on *shotgun* (*shg*, the DE-cadherin homolog of *Drosophila*) and *armadillo* (the *Drosophila* catenin homolog) that interact at the cortex of the cells where they form a complex [18,19]. This adhesion allows proteins produced by somatic cells, such as PIWI, Unpaired and Decapentaplegic [20] to regulate the germline stem cell asymmetric divisions, allowing their self-renewal on the one hand and differentiation of cystoblasts on the other [12,17,21]. In later stages of oogenesis, DE-cadherin is important for the positioning of the oocyte at the posterior pole of the follicle, as well as for the migration of border and centripetal follicle cells within the trophic chamber [11,22]. 

Other cadherins, such as N-cadherin and especially so the non-classical cadherin Cad99C, have been detected by *in situ *hybridization in the ovary of the fruit fly [23]. Cad99C, which is the fly homolog of vertebrate protocadherin 15, is present in microvilli of follicle epithelial cells and appears to play a role in vitelline membrane assembly [24]. There is also evidence that, in addition to DE-cadherin, other cadherins may contribute to the organization of *Drosophila *testis. Furthermore, both DE- and DN-cadherin have been shown to be localized at the interface between the hub and germline stem cells of the embryonic gonad [25]. 

This intricate association between cadherins and different steps of gonad formation and gametogenesis in *Drosophila *males and females prompted us to ask whether the same proteins are players in oogenesis and spermatogenesis in honey bees. The question arose upon considering that the above mentioned differences in ovariole organization among flies and bees and also the differences in spermatogenesis compared to other insect orders [26] may be reflected in divergent cadherin localization and also possible function(s). Hymenopterans, which include the highly eusocial ants, bees and wasps, have a haplodiploid mode of sex determination under the control of an initial molecular switch mechanism [27]. Consequently, hymenopteran males, as the haploid sex, necessarily deviate from other insects in their meiotic division steps. They abort the first reductive division and form only two spermatids, whereby only the larger one is thought to give rise to a functional spermatozoid [28,29,30,31,32].

In a previous study on the honey bee ovary we detected a nuclear localization of a cadherin by means of a heterologous pan-cadherin antibody [33], raising the question as to the possible involvement of cadherins in intracellular signaling pathways and/or regulation of gene expression. As answering this question would require more precise tools than heterologous antibodies, we raised honey bee-specific antibodies to the N- and C-terminal domains of both N- and E-cadherin and compared their subcellular localization in the gonads. Immunofluorescence analysis not only confirmed the nuclear localization of these cadherins but also revealed considerable differences among ovarioles of queens and egg-laying workers, indicating major differences with regard to the molecular architecture of the oogenesis processes in the two female castes. Furthermore, when investigating the localization of these cadherins in testiolar tubules of drone larvae, we denoted a strong association of the C-terminal domain of E-cadherin with the polyfusomes of cystocyte clusters.

## 2. Results and Discussion

An antibody against the C-terminal domain of the honey bee N-cadherin (N-cadC) was generated through cloning and expressing a C-terminal protein fragment in a bacterial expression vector. Further antibodies were raised against KLH-conjugated synthetic peptides representing the N-terminal domain of N-cadherin (N-cadN) and the N- and C-terminal domains of E-cadherin (E-cadN and EcadC). These polyclonal antibodies were validated through western blot analyses. As the antibody against the N-cadN detected two proteins it is possible that both were also seen in the immunofluorescence analyses.

### 2.1. Protein Identification by Western Blot Analysis

**Figure 1:**
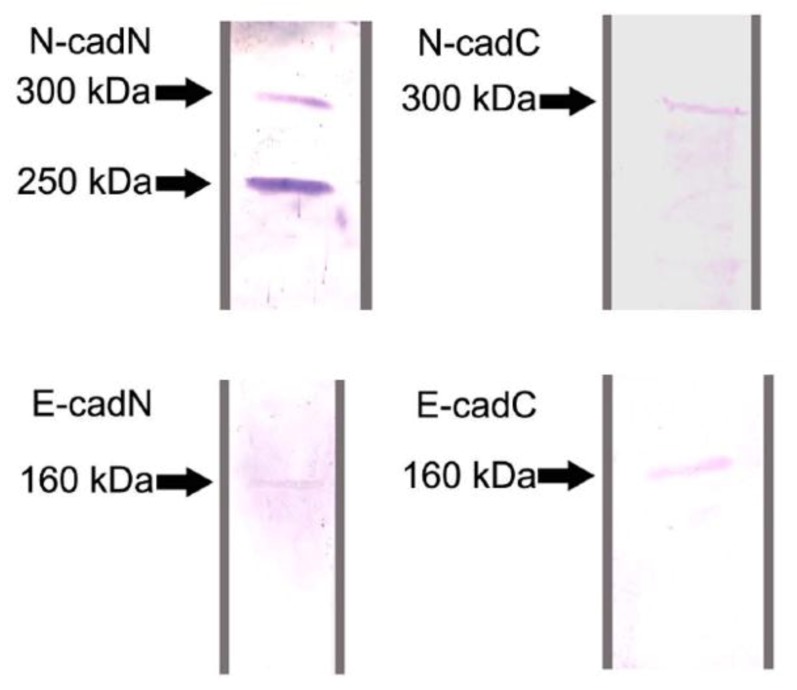
Western blot analyses with antibodies directed against the N- and C-terminal domains of *Apis mellifera* E- and N-cadherin, done on protein extracts of *Apis mellifera* larval testes. A 160 kDa protein was detected by the antibodies raised against the two E-cadherin domains (E-cadN and E-cadC). The antibody raised against C-terminal N-cadherin (N-cadC) detected a protein of approximately 300 kDa, which was also recognized by the antibody against the N-terminal domain (N-cadN), the latter additionally reacting with a protein of approximately 250 kDa.

Western blot analyses were done to characterize and confirm the specificity of the domain-specific anti-cadherin antibodies produced in this study. In blots produced from ovary and testes protein lysates, the N-cadN and N-cadC antibodies both recognized a protein of approximately 300 kDa (Figure 1), which is close to the computationally predicted molecular mass for the honey bee N-cadherin (340 kDa). The antibody against N-cadN strongly reacted with a second band at approximately 250 kDa which may be a cleaved product or an isoform of N-cadherin, as mutually exclusive exons have been found for *Apis mellifera* by comparative genomic analyses against *Drosophila melanogaster* cadherin [34]. At present, there is no information on whether these may actually play different roles in cell-cell interaction or intracellular signaling.

The antibodies generated against the E-cadN and E-cadC domains both reacted with a single protein band of 160 kDa, which is a close match to the genomically predicted molecular mass for E-cadherin (167 kDa). The specific detection of *Apis mellifera* E-cadherin and N-cadherin by these antibodies indicates their usefulness in immunolocalization studies on honey bee tissues.

### 2.2. Differential Localization of Cadherin Domains in Queen and Worker Ovaries Indicates Alternative Functions Related to Fertility Status

#### 2.2.1. Immunolocalization of the N- and C-terminal N-cadherin Domains (N-cadN and N-cadC) in Honey Bee Ovaries

Immunofluorescence analyses were performed on ovarioles of egg-laying queens and adult workers. For the interpretation of the results it is important to keep in mind that the confocal micrographs shown for both N- and E-cadherin are all single optical sections (~1 μm width). We preferred to show single sections instead of 3D stack reconstructions to allow a clear distinction between nuclear and cytoplasmic localization patterns of these cadherins. Such unconventional nuclear localization has already been evidenced in our previous study on honey bee ovaries [33]. 

**Figure 2:**
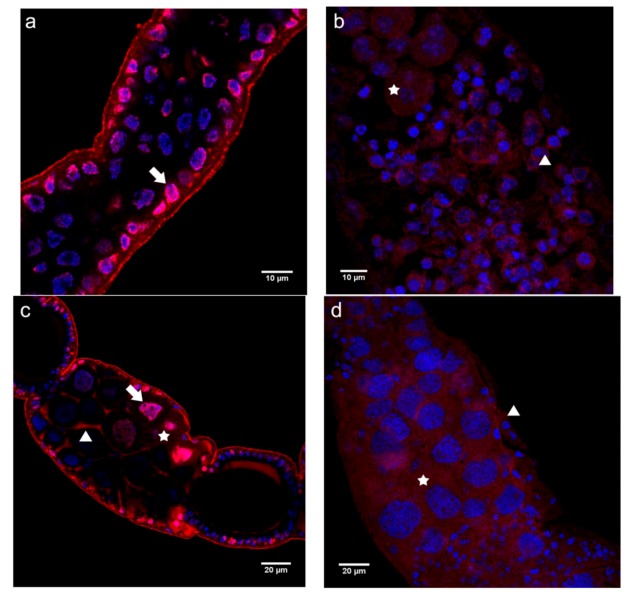
Immunofluorescence analysis with an antibody raised against the N-terminal domain of N-cadherin (N-cadN) performed on ovaries of egg-laying queens (**a** and **c**) and queenless workers (**b** and **d**). Shown are single confocal section images of 1 μm thickness taken in the germarial region and early previtellogenic follicles. (**a**) The germarial region of a queen ovariole showed a strong N-cadN signal in the periphery of the nuclei, in addition to punctate labeling in the more central nuclear positions (arrow). (**b**) The germarium of worker ovarioles showed dispersed N-cadN throughout the cytoplasm (star), as well as diffuse spots within the nuclei (arrowhead) of germline and somatic cells, which cannot be distinguished at this level of analysis. (**c**) Previtellogenic follicles of a queen ovariole revealed N-cadN immunoreactivity in association with plasma membranes (arrowhead) of nurse cells, as well as a nuclear signal (arrow), which became increasingly stronger (asterisk) in nurse cells adjacent to the oocyte (lower right corner, surrounded by a layer of follicle epithelial cells). (**d**) In previtellogenic follicles of worker ovarioles N-cadN immunoreactivity appeared homogeneously dispersed in the cytoplasm and as punctuate signals in the nuclei (arrow) of trophocytes (asterisk) and somatic cells (arrowhead).N-cadN immunoreactivity detected with a Cy3-conjugated secondary antibody is shown in red, nuclei counterstained with Hoechst 33258 appear in blue.

In the germarium of queen ovarioles, the N-cadN domain was primarily detected as dispersed over the nuclei of somatic and germline cells. We did not find significant labeling in the cytoplasm or in association with cell membranes (Figure 2a). This is in contrast to findings for the equivalent region in worker ovarioles, where immunoreactivity to the N-cadN domain antibody was observed in the cytoplasm, as well as in the nuclei of somatic and germline cells, where it was apparent as a punctate pattern (Figure 2b).

In previtellogenic follicles of queens, the nuclear labeling pattern was similar to the one seen in the germarial region (Figure 2c), with trophocytes showing both nuclear and cytoplasmic perinuclear labeling (Figure 2c). In addition, a strong association of the N-cadN domain with cell membranes became apparent in all cells of the trophocytic chamber (Figure 2c). Again, in worker ovarioles the labeling appeared almost homogeneously dispersed over the cytoplasm and focally present in the nuclei of all the cells (Figure 2d), thus differing considerably from queen ovarioles in terms of N-cadN distribution in previtellogenic ovarioles.

**Figure 3:**
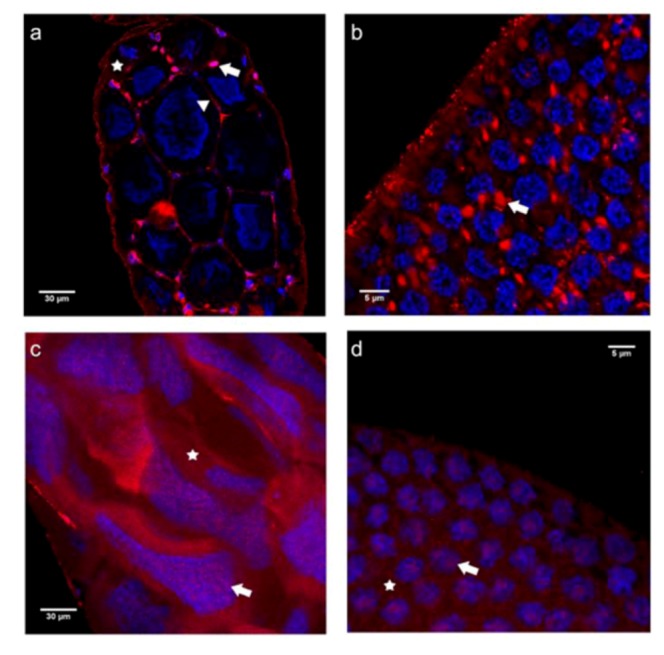
Immunofluorescence analysis with the antibody raised against the N-terminal domain of N-cadherin (N-cadN) performed on ovaries of egg-laying queens (**a** and **b**) and queenless workers (**c** and **d**). Shown are single confocal section images of 1 μm thickness taken on vitellogenic follicles. (**a**) The trophic chambers of vitellogenic follicles in a queen ovariole showed N-cadN in association with plasma membranes (arrowhead) of large nurse cells and a nuclear signal in somatic follicular epithelium cells (arrow). The nuclear and cytoplasmic signals for N-cadN in nurse cells were weak. (**b**) The follicular epithelium covering the oocyte of queen vitellogenic follicles revealed N-cadN agglomerates in the cytoplasm (arrow). (**c**) Trophocytes of vitellogenic follicles of a worker ovariole showed a highly dispersed N-cadN labeling throughout the cytoplasm (asterisk), as well several nuclear foci (arrow) in these germline-derived cells. (**d**) A similar dispersed distribution pattern for this cadherin moiety was also seen in the cytoplasm (asterisk) and nuclei (arrow) of follicular epithelium cells covering oocytes. N-cadN immunoreactivity is shown in red, counterstained nuclei are in blue.

In vitellogenic follicles of queen ovarioles, the N-cadN localization pattern closely resembled that seen in previtellogenic ovarioles, with labeling seen at the cell membranes and scattered throughout the cytoplasm in the nurse cells adjacent to the oocyte (Figure 3a). Plasma membranes and nuclei were also labeled in follicular epithelium cells covering the nurse cell chamber (Figure 3a). In the follicular epithelium surrounding the oocyte, N-cadN immunoreactivity was seen as spots (Figure 3b), these possibly representing adhesion points between these epithelial cells, similar to the adhesion foci between the large nurse cells seen in the trophic chamber (Figure 3a). 

In worker vitellogenic follicles, the staining pattern differed drastically from that seen in queens. Instead of a membrane and nuclear/perinuclear localization, the N-cadN domain appeared to be nearly homogeneously dispersed throughout the cytoplasm of nurse cells, in addition to small dots within the polyploid, elongated or stellate nuclei (Figure 3c). Such a diffuse distribution of N-cadN was also observed in follicular epithelium cells covering the oocyte chamber (Figure 3d).

These results show that queens and workers hold in common a nuclear N-cadN immunoreactivity apparent all along the proximo-distal axis of the ovarioles, inferring that N-cadherin may possibly be involved in the nuclear architecture and/or regulation of gene expression. A nuclear localization of N-cadherin has already been evidenced in vertebrate cells. This, occurring after cleavage of the cytoplasmic domain mediated by ADAM10 and a presenilin/γ-secretase system [35,36], inhibited cell-cell adhesion and the activity of the CBP (CREB-binding protein) transcription factor [37]. In ovaries of *Apis mellifera*, nuclear labeling has already been observed for other cytoskeletal proteins, such as β-tubulin and actin [6], indicating that cytoskeletal proteins may indeed be having alternative roles during gametogenesis. 

The nuclear localization associated with a cytoplasmic signal in previtellogenic and vitellogenic follicles is also indicative of possible signal transduction from the cell membranes to the nuclei, especially so in the cells adjacent to the oocyte. Cytoplasmic transport through the ring canals mediated by cytoskeletal proteins, as observed among trophocytes of *Drosophila* [38], is another hypothesis to be considered.

**Figure 4:**
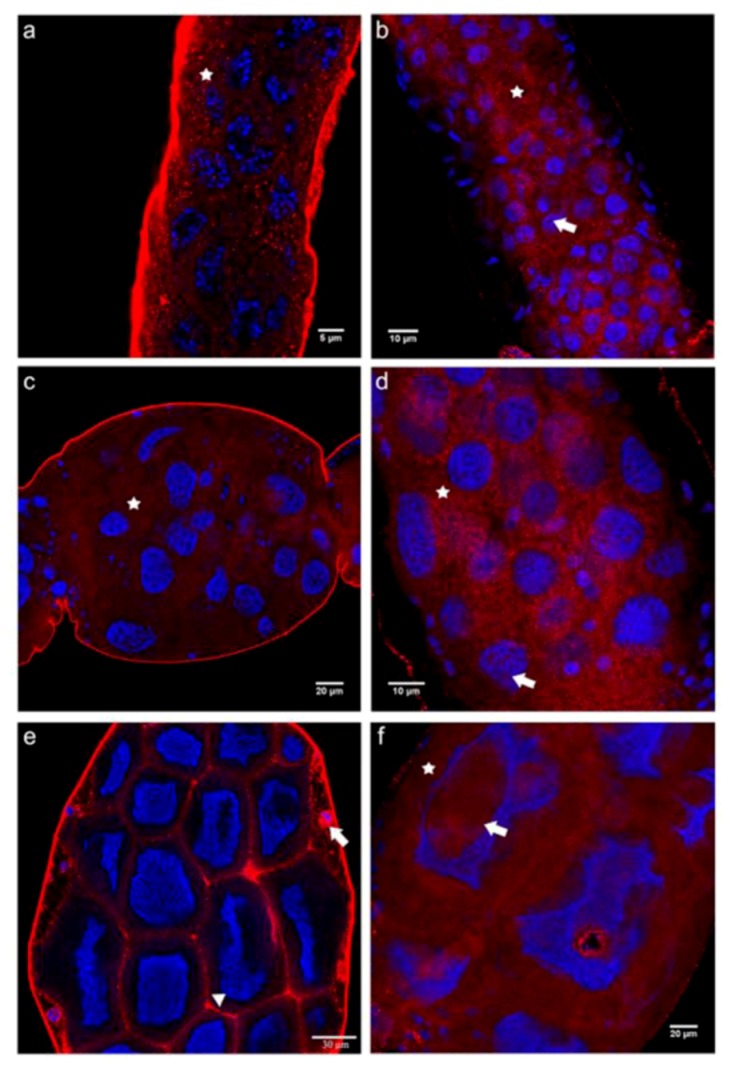
Immunofluorescence analysis with an antibody raised against the C-terminal domain of N-cadherin (N-cadC) performed on ovaries of egg-laying queens (**a**, **c** and **e**) and of queenless workers (**b**, **d** and **f**). Shown are single confocal section images of 1 μm thickness. (**a**) In the germarium of queen ovarioles, punctate labeling was seen throughout the cytoplasm (asterisk), as well as in the nuclei. (**b**) A similar picture as that seen in queens was also found in the cytoplasm (asterisk) and nuclei (arrow) of cells in the germarium of worker ovarioles. (**c** and **d**) Trophocytes of queen and worker previtellogenic follicles showed a highly similar N-cadC labeling pattern in the cytoplasm (asterisk) and in nuclei (arrow). (**e**) In vitellogenic follicles of queen ovarioles, N-cadC appeared associated with the cell membranes of nurse cells (arrowhead), in addition to a strong nuclear signal in follicular epithelium cells (arrow). (**f**) In vitellogenic follicles of worker ovarioles, the distribution of N-CadC immunoreactivity was similar to that seen in previtellogenic follicles, with trophocytes labeled homogeneously all over the cytoplasm (asterisk) and in the nuclei (arrow). N-cadC immunoreactivity is shown in red, counterstained nuclei are in blue.

The distribution pattern of the C-terminal domain of N-cadherin differed considerably from that seen for the N-terminal one. Starting from the germarium down to previtellogenic follicles, N-cadC immunoreactivity showed a rather homogeneous cytoplasmic distribution in both queen and worker ovarioles (Figure 4a–d). While this pattern also persisted in the trophic chambers of vitellogenic follicles in the worker caste (Figure 4f), a membrane association of the N-cadC domain was discernible in the corresponding region of queen ovarioles (Figure 4e).

The fact that only the N-cadN but not the N-cadC domain was detected in the nuclei of germline cells in queen ovariole indicates that N-cadherin may have undergone proteolytic cleavage, and that these domains may play different roles. Notwithstanding, in worker ovarioles we observed the same distribution pattern for the N-cadC and the N-cadN domains, and the number of nuclear foci in trophocytes increased for both domains, probably accompanying endomitotic polyploidization in these cells [9].Taken together, this leads us to infer that the two N-cadherin domains not only play different roles during the oogenesis process in queens, but also that N-cadherin functions may actually differ in this process between the two female castes.

#### 2.2.2. Immunolocalization of the N- and C-terminal E-cadherin Domains (E-cadN and E-cadC) in Honey Bee Ovaries

**Figure 5:**
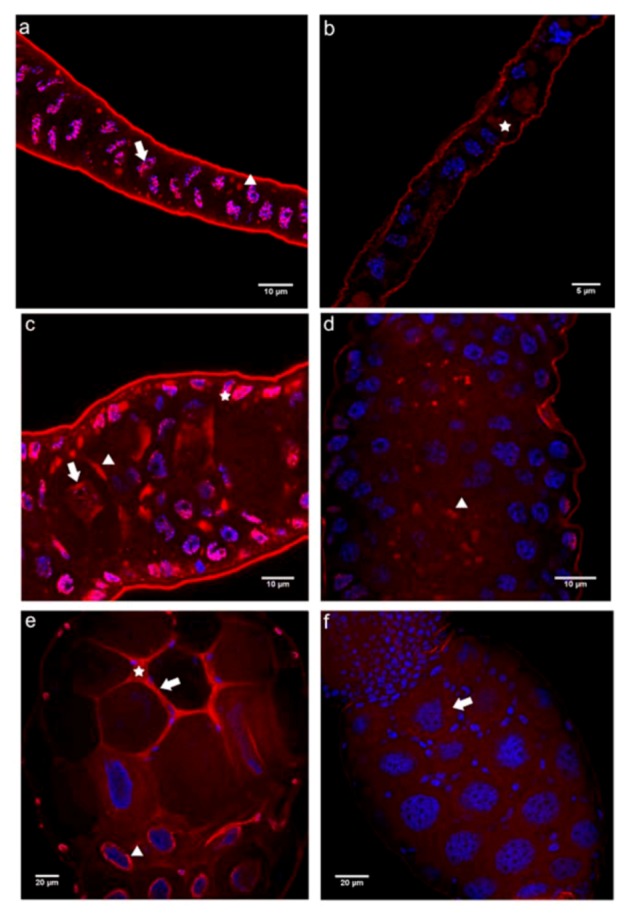
Immunofluorescence analysis with an antibody raised against the N-terminal domain of E-cadherin (E-cadN) performed on ovaries of egg-laying queens (**a**, **c** and **e**) and queenless workers (**b**, **d** and **f**). Shown are single confocal section images of 1 μm thickness. (**a**) E-cadN labeling in terminal filament cells of a queen ovariole appeared scattered throughout the nuclei (arrow) and clustered in certain regions of the cytoplasm (arrowhead). (**b**) In the terminal filament of worker ovarioles, E-cadN labeling was also apparent as cytoplasmic clusters (asterisk), but less so in nuclei. (**c**) In early previtellogenic follicles of queens, strong labeling was seen in the oocyte nucleus (arrow), as well as in the nuclei of trophocytes and follicular epithelium cells (asterisk). Cell-membrane-associated labeling was seen in oocytes (arrowhead). (**d**) In previtellogenic follicles of workers, E-cadN appeared dispersed throughout the cytoplasm of trophocytes and follicular epithelium cells, while cytoplasmic agglomerates were visible in oocytes (arrowhead). (**e**) In vitellogenic follicles of queen ovarioles, E-cadN became localized to the plasma membrane (arrow), but maintained its more homogeneous distribution in the cytoplasm of follicular cells (asterisk). Trophocytes located closer to the oocyte (basal to this image) were intensely labeled in the perinuclear region (arrowhead). (**f**) Trophocytes in the nurse chamber of vitellogenic follicle in worker ovarioles showed a dispersed to slightly clustered E-cadN labeling in the cytoplasm (arrow). E-cadN immunoreactivity is shown in red, counterstained nuclei are in blue.

When assaying the localization of the N-terminal domain of E-cadherin (E-cadN), a picture resembling that of N-cadherin became apparent. In queen ovarioles (Figure 5a,c,e), the E-cadN domain showed strong nuclear and perinuclear signals in both somatic and germline cells in the terminal filament/germarium transition zone (Figure 5a), as well as in the lower germarium where oocytes and nurse cells are already clearly distinguishable (Figure 5c). The same was observed in the trophic chamber of vitellogenic follicles (Figure 5e), here especially in the smaller nurse cells adjacent to the egg chamber. Furthermore, a gradual trend towards cell membrane association of the extracellular E-cadherin domain became visible in oocytes (Figure 5c, arrowhead) and, especially so, in large trophocytes and follicle epithelial cells interspersed among trophocytes (Figure 5e).

As this perinuclear signal was strongest in trophocytes situated closest to the egg chamber, it is possible that E-cadN may be involved in the dumping process of the nurse cell cytoplasm. In this context, E-cadherin may interact with other cytoskeletal proteins to retain the trophocyte nuclei within the remains of the trophocytes, as they discharge their cytoplasm content into the oocyte at the end of oocyte growth [39].

Again, the pattern of E-cadN localization found in queens is in contrast to the one found in worker ovarioles (Figure 5b,d,f). In the latter, E-cadN was primarily seen as dispersed throughout the cytoplasm of terminal filament cells (Figure 5b), and as forming aggregates in the cytoplasm of oocytes situated in the lower germarium (Figure 5d, arrowhead). Such diffuse distribution was also denoted in trophocytes and in the interspersed follicular epithelium cells of vitellogenic follicles (Figure 5f).

**Figure 6:**
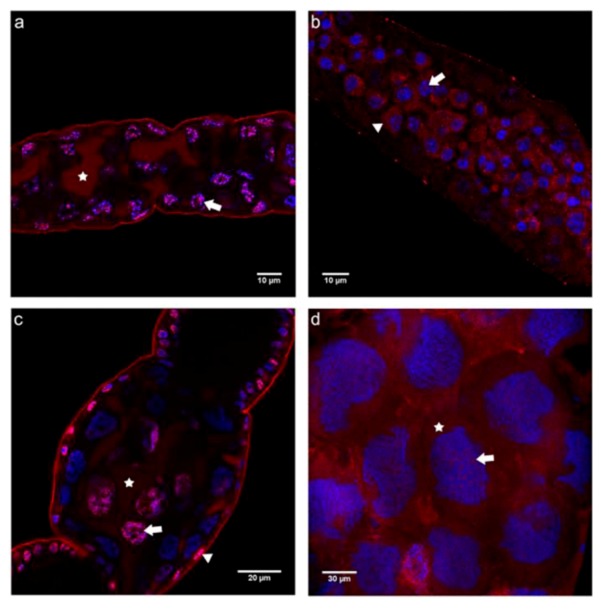
Immunofluorescence analysis with an antibody raised against the C-terminal domain of E-cadherin (E-cadC) on ovaries of egg-laying queens (**a** and **c**) and queenless workers (**b** and **d**). Shown are single confocal section images of 1 μm thickness. (**a**) Germarium of queen ovariole showing homogeneous, extensive cytoplasmic labeling in the periphery (asterisk) and strong foci in the nuclei (arrow). (**b**) An almost complete inversion image to that seen in queens was apparent in the germarium of worker ovarioles, with E-cadC dispersed in the perinuclear cytoplasm (arrowhead) and as smaller dots in the nuclei (arrow). (**c**) In the trophic chamber of previtellogenic follicles in queen ovarioles, nurse cells continued to show the diffuse E-cadC localization in the peripheral cytoplasm (asterisk), as well as maintaining the strong nuclear signal (arrow), especially so in those closer to the oocyte (partially apparent at lower left side). (**d**) Trophocytes of previtellogenic worker follicles continued to show a dispersed distribution of E-cadN throughout the cytoplasm (asterisk), but with a tendency to become more concentrated in the cortex. The nuclei showed innumerous E-cadC dots (arrow). E-cadC immunoreactivity is shown in red, counterstained nuclei are in blue.

Interestingly, the cytoplasmic E cadherin domain (E-cadC) showed a distribution pattern much similar to that of the extracellular domain of this cadherin moiety. In queen ovarioles, a strong nuclear/perinuclear association was visible in the germarium and also in previtellogenic follicles (Figure 6a,c), the only major difference with respect to E-cadN being its diffuse distribution, which apparently extended across neighboring cells (marked by asterisks in Figure 6a,c). In worker ovarioles, immunofluorescence detection with the E-cadC antibody showed diffuse cytoplasmic labeling in the germarium and also in nurse cells of previtellogenic follicles, as well as punctuate labeling associated with nuclear material (Figure 6b,d). 

The E-cadC domain detected within nuclei as well as in association with cell boundaries in ovarioles of queens indicates that it may have both a cell adhesion function and a role in nuclear organization. A nuclear localization has already been described for vertebrate E-cadherin, this being dependent on cleavage of the cytoplasmic domain by a presenilin/γ-tubulin complex, which affects the stability of cellular adhesion by modulating a signaling pathway mediated by the p120catenin-Kaiso complex [40,41,42]. Although there is currently no direct evidence that *Apis mellifera* E- or N-cadherins are cleaved, this could explain certain differences in domain localization observed with the domain-specific antibodies, mainly in the distinct nuclear labeling patterns. Furthermore, as cytoplasmic cleavage has been proven for vertebrate E-cadherin [40,41,42], the hypothesis is certainly plausible.

### 2.3. Cadherin Immunolocalization in Testes of *Apis mellifera* Drones - Similarities and Differences between the Sexes

Since the general architecture of insect ovarioles and testiolar tubules is strikingly similar, with oogenesis (in meroists) and spermatogenesis being events that both involve rosette-like cluster formation of germline cells in the early stages of these processes, we decided to also apply the domain-specific cadherin antibodies to the male sex of the honey bee. To do so, we dissected testes of drone larvae in a developmental stage where both mitotic and meiotic divisions still occur in the different regions of the testiolar tubules. 

**Figure 7:**
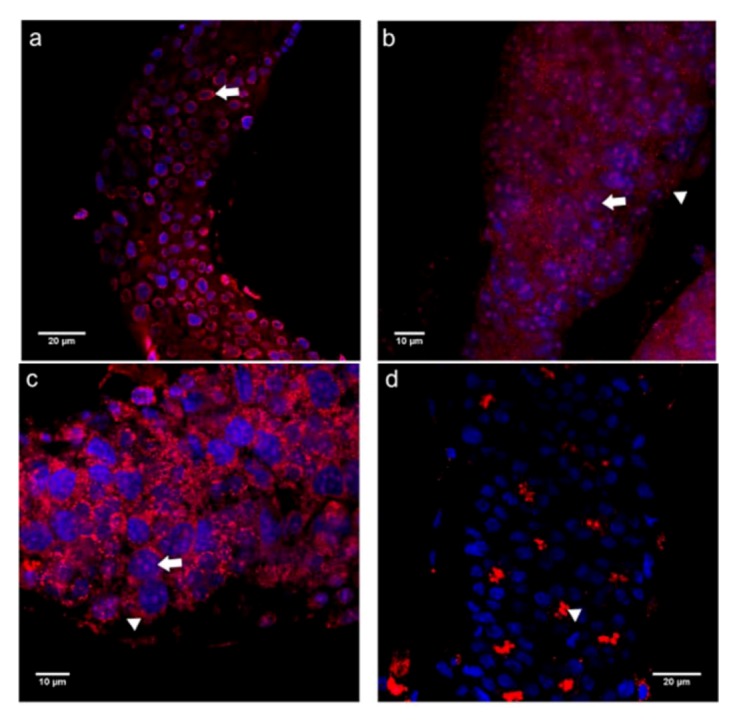
Immunolocalization performed on testes of drone larvae with antibodies raised against the N- and C-terminal domains of E- and N-cadherin. Shown are single confocal section images of 1 μm thickness. (**a**) N-cadN appeared more concentrated in the perinuclear region (arrow) of all cells along the testioles. (**b**) In the basal region of testioles, N-cadC appeared dispersed throughout the cytoplasm (arrowhead) and as large nuclear foci (arrow). (**c**) In the basal region of testioles, E-cadN was detected in the cytoplasm (arrowhead) and as nuclear foci (arrow) in all cells. (**d**) E-cadC was detected in the center of cystocyte rosettes in colocalization with the fusomes (arrowhead). Cadherin immunoreactivity is shown in red, counterstained nuclei are in blue.

For both cadherins, however, we noted clear differences between ovariolar and testiolar distribution of these cell adhesion proteins. Neither with the N- nor the E-cadherin antibodies did we find evidence for a membrane association in testiolar tubules (Figure 7). The closest resemblance to cadherin localization seen in ovarioles was found for the N-cadN domain which showed a strong perinuclear signal in germline cells (Figure 7a). Immunoreactivity representing the N-cadC (Figure 7b) and the E-cadN (Figure 7c) domains appeared dispersed throughout the cytoplasm and with strong intranuclear foci. The most striking and unique feature seen in testioles, however, was the prominent signal for the E-cadC domain within the polyfusomes of spermatogonial clusters (Figure 7d), indicating a strong association of this domain with cytoskeletal elements present in these intercellullar bridges. 

The perinuclear N-cadN localization seen throughout the tubules is similar to the pattern observed in queen ovarioles. This suggests that the domain may not be involved in cellular adhesion but instead co-localizes with parts of the endoplasmic reticulum. The N-cadC distribution, on the contrary, was more like that seen in the worker ovarioles, with concentrated nuclear foci in all cells. Drones and workers also shared the cytoplasmic labeling for E-cadN, except that in testioles the nuclear foci were present in both somatic and germ cells. A major difference between the two sexes was, however, denoted for E-cadC, which in testioles turned out to be associated with the polyfusome fusome, this being a germline-specific organelle consisting of a complex arrangement of cytoskeletal proteins in the center of the cystocyte rosettes [43]. 

## 3. Experimental Section

### 3.1. Bees

Adult virgin queens were reared following standard apicultural methods and collected at emergence from the brood cells. They were then kept in JZBZ cages for 15 days within a colony to undergo maturation. Egg-laying queens were directly retrieved from their hives. Adult workers with active ovaries were obtained from orphaned hives. 

Last instar drone larvae were retrieved from brood frames. This larval stage was chosen for cadherin localization because it best shows all the different meiotic phases of spermatogenesis, which occurs during the late larval and early pupal stages in *Apis mellifera* development [4,29,30].

### 3.2. Honey Bee E- and N-cadherin Gene Predictions

Genes encoding E- and N-cadherin orthologs were identified by BLASTP searches using *Drosophila melanogaster *cadherins as queries against the set of predicted honey bee gene products (Amel_pre_release_2_OGS_pep) [44]. The genomic scaffold coordinates obtained by TBLASTN searches against the honey bee genome (version Amel v 4.0) [44] served as input for manual exon annotation by using an Artemis version 7.0 platform [45] run on a LINUX server.

Molecular mass prediction of N-cadherin cytoplasmic (N-cadC) domain was accomplished by means of the Compute pI/Mw program of ExPASy [46]. The predicted molecular mass value for the N-cadC fragment expressed in bacteria was 13.6 kDa, this taking into account the 22 amino acids present in the expression clone that also introduced a tag of six histidine residues.

### 3.3. RT-PCR Analysis

Total RNA was extracted using TRIzol reagent (Invitrogen) according to the manufacturer’s instructions. Samples were treated with DNase before spectrophotometric determination of RNA quality and quantity. First-strand cDNA synthesis was done with 2 μg of each RNA sample in an oligo(d)T_12-18_ (Invitrogen) primed reaction with SuperScript II (Invitrogen) reverse transcriptase. 

### 3.4. *In vitro* Expression of the N-cadherin Cytoplasmic Domain

For *in vitro* expression of the N-cadherin cytoplasmic domain (amino acid position 2944 to 3049 in the predicted protein GB12853-PA, Official Gene Set 2) we used the Gateway Technology with a Clonase II system (Invitrogen). Specific primers were designed to amplify the respective cDNA fragment including the *attB* site used to introduce the fragment into the entry clone of the Gateway system. 

The first step was a reaction with specific primers to amplify a fragment that contains the *attB* site responsible for recombination. This reaction was a touchdown PCR performed with Platinum *Taq* DNA polymerase High Fidelity (Invitrogen) under the following conditions: 94 °C for 2 min, 15 cycles of 94 °C for 30 s with a touchdown series of annealing cycles (3 cycles at 60 °C for 30 s, 3 cycles at 59 °C for 30 s, 3 cycles at 58 °C for 30 s, 3 cycles at 57 °C for 30 s, 3 cycles at 56 °C for 30 s) each with an elongation step at 68 °C for 30 s, then 15 cycles at 94 °C for 30 s, 55 °C for 30 s, 68 °C for 30 s, and a final elongation step at 68 °C for 7 min. Aliquots of this amplification were used for a second PCR step with primers ATTB F and ATTB R in order to select for the sequences with the *attB* site at their extremities. The second reaction was run under the same conditions as the first one and the products of both amplifications were electrophoretically separated in 1% agarose gels and stained with ethidium bromide.

The amplified fragments were purified by means of the Perfectprep Gel Cleanup kit (Eppendorf) and then quantified to initiate the BP reaction which involves a recombination between the *attB* site of the PCR fragment and the *attP* site of the entry clone. The reaction was performed with 4 μL of TE buffer (Tris-HCl 100 mM, EDTA 1 mM, pH 8.0), 3 μL of the purified PCR product (120–150 ng), 1 μL of pDONR 221 clone (150 ng/μL) (Invitrogen) and 2 μL of BPclonase II enzyme (Invitrogen). The reaction occurred at 25 °C for 1 h, and 1 μL of the solution was used to transform chemo-competent DH5α *E. coli *cells. Clones were selected with kanamycin (0.1 mg/mL) in LB agar medium (USB).

In order to validate the orientation of the cloned N-cadherin C-terminal domain fragment, the latter was sequenced by using a BigDye Terminator v3.1 Cycle Sequencing system (Applied Biosystems) and universal sense and antisense M13 primers in combination with gene-specific sense and antisense primers. Sequencing was done on automatic sequence analyzers (ABI 310 and ABI 3100, Applied Biosystems).

An entry clone was used for the LR reaction which consists of a recombination between the *attL* site obtained through the BP reaction and the *attR* site of the pDEST^TM^ 17 expression vector (Invitrogen). Experimental conditions and reagents used were the same as those described for the BP reaction, except for the use of ampicillin (0.1 mg/mL) in the selection of transformants. Occurrence of the LR reaction was verified after plasmid extraction by means of PCR reactions with a universal T7 primer and the specific antisense primer.

Chemo-competent *E. coli*, BL21 (DE3) Rosetta clones, previously transformed with expression vectors, were selected with ampicillin and cloramphenicol (0.1 mg/mL) and pre-inoculated for 16 h at 37 °C (180 rpm). After this period, a pre-inoculum was diluted in liquid LB medium containing the same antibiotics and maintained at 37 °C (250 rpm). Once it reached an O.D. of 0.4–0.6, a 2 mL aliquot of the culture was collected, serving as T_0_ expression reference. Protein expression in the remaining cells was induced by addition of IPTG (200 mM) to the culture medium. The induction was monitored hourly by collecting 2 mL aliquots of the *E. coli *culture collection until 4 h after IPTG addition. The samples were centrifuged and the pellets re-suspended in lysis buffer (NaCl 300 mM, Tris-HCl 50 mM, pH 8.0, supplemented with 0.2 mg/mL of lysozyme, 2 mM MgCl_2_, 20 mM imidazol, 20 mM β-mercaptoethanol, 2% of Tween-20, 12% glycerol). Cells were lysed by sonication or heat shock (10 × 30 s at 42 °C followed by 30 s in liquid nitrogen). After lysis, the fragments were centrifuged (20 min at 4 °C, 3,000× *g*), supernatant was collected and the pellet re-suspended in lysis buffer. Aliquots of these were separated by SDS-PAGE (12%) to detect the expression of the expected N-terminal C-cadherin (N-cadC) fragment. 

The purification of N-cadC was done by affinity chromatography using a Ni Sepharose 6 Fast Flow (GE Healthcare) matrix following manufacturer’s instructions. The peptide concentration in the elution volume was quantified by means of the bicinchoninic acid (BCA) method [47] with bovine serum albumin (BSA) serving as standard.

In order to confirm fragment identity, an aliquot was analyzed by SDS-PAGE on a 12% gel. The band corresponding to the peptide was cut out and treated with acetonitrile for peptide sequence analysis by mass spectrometry in a MALDI TOF/TOF—AXIMA Performance system (Shimadzu Biotech).

### 3.5. Synthetic Peptides

Peptides corresponding to the extracellular domains of E-cadherin (E-cadN) and N-cadherin (N-cadN), as well as the E-cadherin cytoplasmic domain (E-cadC) were commercially commissioned (GenScript Corp.) to contain an extra cystein residue for keyhole limpet hemocyanin (KLH) conjugation. The peptide sequences were as follows: N-cadN KEGVRVVTARPLDREC, E-cadN DNPDNGGTITYSFVTC, E-cadC PVGMKTRGSDEVPDIC. 

### 3.6. Antibody Production

Before the first intradermic injection, 10 mL of blood were collected from the ear vein of New Zealand albino rabbits to serve as pre-immune serum controls. For the intradermic injections, peptide aliquots of 100 μg each were diluted in 400 μL of PBS (100 mM, pH 7.4) and mixed with 500 μL of Freund’s complete adjuvant. Twenty days after this first injection, a boost injection was given with the same peptide quantity mixed with Freund’s incomplete adjuvant, and after another 15 days, blood was collected from the ear vein. For serum separation, the blood samples were left at room temperature for 2 h for clotting and then centrifuged at 2,000× *g*, the sera were collected and, after adding sodium azide (0.02 M), stored at −20°C.

In order to purify the antibodies, 100 μL of the synthetic peptides were dot blotted to a nitrocellulose membrane (Amersham Biosciences). The blots were cut into small pieces, added to the respective serum and incubated for 3 h at room temperature. Subsequently, the membranes were removed and washed six times with TBS-Tween (Tris 2 mM, pH 7.4, NaCl 500 mM, Tween 20 0.05%) before eluting the antibodies with triethylamine solution (1.4%). The eluted antibodies were dialyzed in two incubation steps against TBS-Tween with sodium azide (0.1%). 

### 3.7. Dot Blot and Western Blot Analyses

The collected sera and purified antibodies were tested first by dot blot analysis against the respective synthetic peptides and gonadal protein extracts. After blotting the test peptides for 15 min, the nitrocellulose membranes (Amersham Biosciences) were stained with Ponceau S (0.6%) to assess blotting efficiency. Unspecific binding sites were then blocked with a solution of 10% dried skimmed milk (Nestle Molico) diluted in TBS-Tween (Tris-HCl 50 mM, pH 8.5, Tween 0.5%, NaCl 0.03%) for 1 h at room temperature. The membranes were incubated for 1 h at room temperature with the primary antibodies diluted 1:1000 in the blocking solution. After two washes in TBS-Tween, the membranes were next incubated in an alkaline phosphatase-conjugated polyclonal goat anti-rabbit IgG secondary antibody (Santa Cruz) diluted at 1:5000 in TBS-Tween. Immunodetection was done with NBT (USB) and BCIP (USB) diluted in TBS-Tween.

SDS-PAGE analyses followed a standard protocol [48]. For N-cadC expression analysis, 10 μL aliquots of each of the IPTG induction monitoring samples were separated on 12% or 14% SDS-polyacrylamide gels, followed by silver or Coomassie staining 

For western blot analyses, ovary and testis protein homogenates separated in 6% or 7.5% SDS-polyacrylamide gels were transferred to nitrocellulose membranes (Amersham Biosciences). These were first stained with Ponceau-S and de-stained before blocking unspecific sites with dried skimmed milk diluted in TBS-Tween for 16 h at 4 °C. Membranes were incubated for 2 h with the different primary antibodies diluted in blocking solution at the following concentrations: Anti-N-cadC 40 μg/mL, Anti-N-cadN 100 μg/mL, Anti-E-cadC 12 μg/mL, Anti-E-cadN undiluted and Anti-histidine (Sigma) 1:1000. After four washes in TBS-Tween, the membranes were incubated with secondary antibodies for 1 h. The peroxidase-conjugated secondary antibody used for anti-histidine detection (Sigma) was diluted 1:12000 in PBS-Tween (0.1 M, pH 7.2, 0.05% Tween 20). The detection process was done by using the ECL Western Blot Analysis System (Amersham Biosciences) and exposure to autoradiographic film (Hyperfilm MP, Amersham Biosciences). Alternatively, proteins reacting with the antibodies were detected through NBT (USB) and BCIP (USB) chromogenic reactions.

### 3.8. Cadherin Immunolocalization in *Apis mellifera* Gonads

Drone larvae, adult queens and workers were dissected in Ringer and the gonads were removed. Ovarioles and testioles were individualized in culture medium specifically designed for *Apis mellifera* larvae [49] and subsequently fixed in paraformaldehyde (4%) for 1 h at room temperature. After fixation, samples were incubated in glycine (0.1 M diluted in PBS) and permeabilized in PBS-T (Triton X-100 2%). Blocking of unspecific binding sites was done by immersion in PBS-BSA (1%) for 1 h. Ovarioles and testioles were incubated for 16 h at 4 °C with primary antibodies at the following protein concentrations: Anti-N-cadC 10-30 μg/100 μL, Anti-N-cadN 7–9.5 μg/100 μL, Anti-E-cadC 3.75–25 μg/100 μL, Anti-E-cadN 2–20 μg/100 μL.

After washing in PBS and PBST, they were incubated with a Cy3-conjugated F(ab') fragment of a polyclonal sheep-anti-rabbit IgG antibody (Sigma) diluted 1:200 in PBST-BSA (0.1%). Incubation with the secondary antibody was done at room temperature over 4 h. Nuclei were counterstained with Hoechst 33258 (0.25 mg/mL in PBST) for 30 min. After washing in PBS, the samples were prepared as whole mounts in undiluted glycerol and analyzed by confocal microscopy (Leica TCS-SP5 System). Artwork was created with Adobe Photoshop 7.

## 4. Conclusions

By employing domain-specific antibodies generated against honey bee E- and N-cadherin, we could show that these proteins exhibit a prominent nuclear and cytoplasmic localization in gonads of both sexes and castes, which indicates that they may have alternative functions besides their expected role in cellular adhesion. Such functions may possibly be associated with signaling pathways. 

On the one hand, these results confirmed the peculiar phenomenon of a nuclear localization of cadherin in honey bee ovaries, previously detected with heterologous antibodies [33]. On the other, they denote apparently major differences with respect to caste in terms of subcellular localization of the two cadherins, thus providing novel insights concerning their possible functions in the organization of the honey bee ovary. Such pronounced differences in the molecular architecture of queen *versus* worker ovarioles are described here for the first time and were not apparent in immunofluorescence analyses of the ovarian microfilaments and tubulin cytoskeleton [6]. We infer that the differences seen in cadherin localization may be related to the dynamics of certain steps in oogenesis, setting the fast and high-throughput egg production in queens apart from the spurious egg production in workers, which is furthermore diminished through social control via pheromones and worker policing [50,51,52].

Furthermore, when assaying larval drone gonads we observed a peculiar association of the E-cadC domain with polyfusomal proteins in the testiolar tubules, this certainly deserving further investigation, considering the abnormal meiosis steps in the haploid male sex. 
